# Biphasic Salt Effects on *Lycium ruthenicum* Germination and Growth Linked to Carbon Fixation and Photosynthesis Gene Expression

**DOI:** 10.3390/ijms26157537

**Published:** 2025-08-04

**Authors:** Xinmeng Qiao, Ruyuan Wang, Lanying Liu, Boya Cui, Xinrui Zhao, Min Yin, Pirui Li, Xu Feng, Yu Shan

**Affiliations:** 1 Jiangsu Key Laboratory for the Research and Utilization of Plant Resources, Institute of Botany, Jiangsu Province and Chinese Academy of Sciences, Nanjing 210014, China; xinmeng_qiao@163.com (X.Q.); 15366627699@163.com (R.W.); fengxu@cnbg.net (X.F.); 2 National Wolfberry Engineering Research Center, Institute of Wolfberry Engineering Technology, Ningxia Academy of Agriculture and Forestry Sciences, Yinchuan 750002, China; syndia1980@126.com

**Keywords:** salt stress, germination, photosynthesis, transcriptome, carbon fixation of Calvin cycle

## Abstract

Since the onset of industrialization, the safety of arable land has become a pressing global concern, with soil salinization emerging as a critical threat to agricultural productivity and food security. To address this challenge, the cultivation of economically valuable salt-tolerant plants has been proposed as a viable strategy. In the study, we investigated the physiological and molecular responses of *Lycium ruthenicum* Murr. to varying NaCl concentrations. Results revealed a concentration-dependent dual effect: low NaCl levels significantly promoted seed germination, while high concentrations exerted strong inhibitory effects. To elucidate the mechanisms underlying these divergent responses, a combined analysis of metabolomics and transcriptomics was applied to identify key metabolic pathways and genes. Notably, salt stress enhanced photosynthetic efficiency through coordinated modulation of ribulose 5-phosphate and erythrose-4-phosphate levels, coupled with the upregulation of critical genes encoding RPIA (Ribose 5-phosphate isomerase A) and RuBisCO (Ribulose-1,5-bisphosphate carboxylase/oxygenase). Under low salt stress, *L. ruthenicum* maintained intact cellular membrane structures and minimized oxidative damage, thereby supporting germination and early growth. In contrast, high salinity severely disrupted PS I (Photosynthesis system I) functionality, blocking energy flow into this pathway while simultaneously inducing membrane lipid peroxidation and triggering pronounced cellular degradation. This ultimately suppressed seed germination rates and impaired root elongation. These findings suggested a mechanistic framework for understanding *L. ruthenicum* adaptation under salt stress and pointed out a new way for breeding salt-tolerant crops and understanding the mechanism.

## 1. Introduction

Soil salinity is a huge challenge for agriculture and the environment, affecting a certain percentage of the world’s agricultural land [1]. This widespread issue poses a significant threat to global food security, yet its severity is often underestimated. Understanding how salinity stress impacts plant germination is essential for mitigating its effects and preserving valuable farmland. Selecting an appropriate plant for investigation requires prioritizing salt tolerance alongside economic value.

*Lycium ruthenicum* Murr. (*L. ruthenicum*), commonly known as black wolfberry, thrives naturally in the saline deserts of north-west China and therefore offers an exceptional model for studying salt tolerance [2]. Its natural adaptation to high-salinity environments makes it a promising candidate for salinity-related research. The study by Qin et al. demonstrated that *L. ruthenicum* can tolerate salt concentrations of 150 mM and higher, although both root and leaf tissues are still affected by salt stress [1]. Interestingly, previous studies have reported that low-salt treatments can promote growth in *Arabidopsis thaliana* (L.) Heynh and *Raphanus sativus L. var. longipinnatus* Bailey [3,4]. We observed a similar growth-promoting effect under mild salt stress in *L. ruthenicum* during germination. However, most existing research on this species has focused on metabolite accumulation in ripening fruit [5]. Notably, research has identified structural genes (e.g., *PAL*, *C4H*, *4CL*, *CHS*, *F3′H*, *F3′5′H*, and *UFGT*) and transcription factors such as *bHLH*, *HSF*, *NAC*, *WRKY*, *bZIP*, and *MADS* involved in anthocyanin biosynthesis [6]. Studies on related *Lycium* species provide additional insights. For instance, in *L. barbarum* L. fruit, salt-induced ROS are countered through two complementary strategies: the transcriptional upregulation of flavonoid-glycosylation and carotenoid-esterification genes, as well as enhanced oxidoreductase activity [7]. Whether similar mechanisms operate during early seedling establishment in *L. ruthenicum* remains unknown.

Across other taxa, research has tended to spotlight single genes and microRNAs, such as VvaASMT1 [8], such as OsACA6 in *Oryza sativa* L. [9] and microRNA408 in *Salvia miltiorrhiza* Bunge [10]. Few have explored the comprehensive metabolic changes during physiological processes. Some identified biomarkers [11,12,13,14], such as alkaline phosphatase, lipase, esterase, β-galactosidase, and trypsin, have been noted in *Solanum lycopersicum* L. [15]. Broad-scale metabolomic or transcriptomic surveys have been confined largely to mature organs [7,9,16,17,18], highlighting pathways such as secondary metabolism, purine turnover, pyruvate processing, pentose and glucuronate interconversions, and downstream energy fluxes [19,20]. Moreover, salt stress also led to oxidant damage caused by water deficit and increased ion uptake [21,22]. These findings suggest complex, hormone-mediated processes that require further investigation.

Seed germination, the developmental window most vulnerable to salinity [23,24,25], offers an opportunity to uncover key genes and metabolites involved in early salt tolerance. Previous research has observed that *L. ruthenicum* roots turn purple under salt stress, suggesting Na^+^/K^+^ imbalance [26,27]. Flavonoid glycosylation and carotenoid biosynthesis may play crucial roles in this response, offering potential for genetic improvement and economic benefits [26,28]. Accordingly, the present study integrates transcriptomic and metabolomic profiling with physiological assays to investigate how *L. ruthenicum* seeds perceive and respond to salinity. The aim is to clarify the mechanisms underlying salt tolerance during germination and to inform the development of cultivars that combine robust early growth with desirable metabolic traits.

## 2. Results

### 2.1. Effects of Different NaCl Concentrations on the L. ruthenicum Seeds

To verify the existence of this phenomenon, which salinity stress could affect the growth and development of *Lycium ruthenicum*, it was selected as an experimental material. Moreover, we selected 0, 50 [29], and 150 mM [1] as the experimental concentrations, which we designated as the CK, L, and H groups, respectively. The lower concentration can make sure the photosynthesis will not be weakened; however, a higher concentration could impose substantive stress on the plant without being lethal.

**Figure 1:**
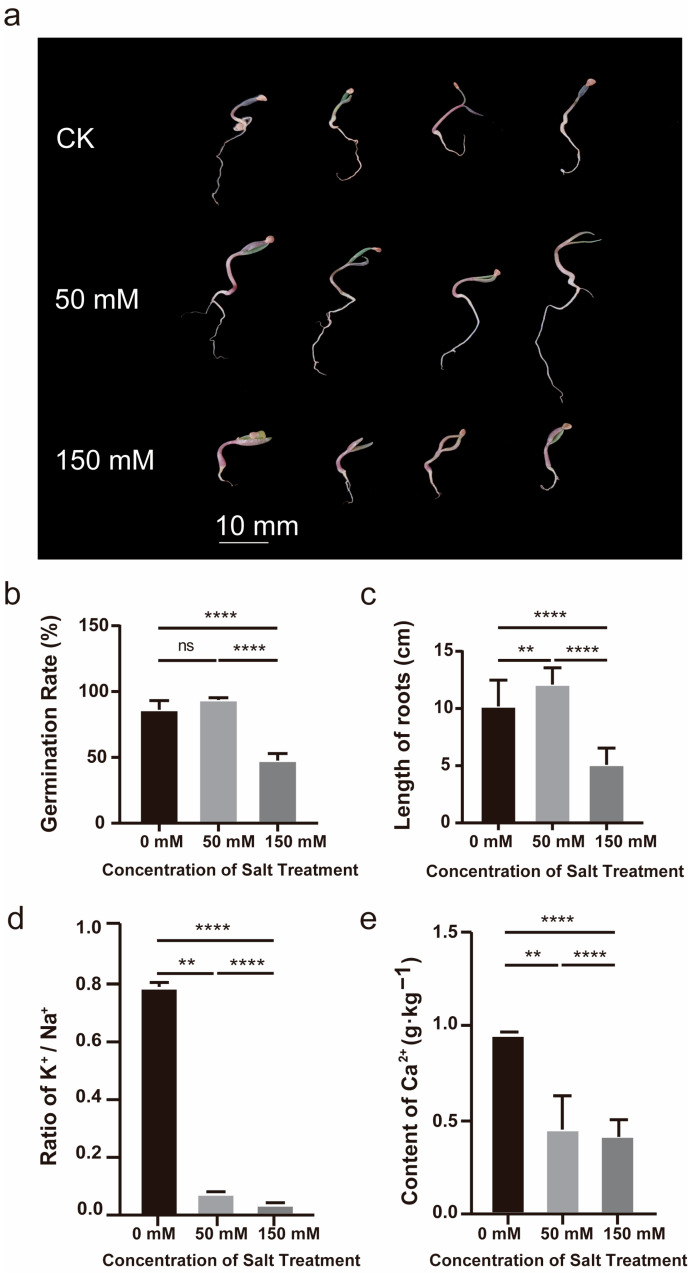
Impact of differential salinity treatments on *L. ruthenicum* seedling growth: (**a**). Phenotype of *L. ruthenicum* seedlings; (**b**). Seed germination rate of *L. ruthenicum* plants; (**c**). Root length of *L. ruthenicum* plants; (**d**,**e**) Ion content of *L. ruthenicum* seeds. At least three replicates were performed. Vertical bars indicate ± SDs of mean. We use asterisks to indicate statistical significance (** *p* < 0.01, **** *p* < 0.0001, ns *p* > 0.05, *n* = 3).

As shown in Figure 1, both the germination rate and root length of the H group were notably inhibited, with the roots exhibiting a distinct purple coloration. High salt stress significantly decreased root length compared to control plants (Figure 1c), clearly demonstrating that severe salinity stress inhibits the growth and development of *L. ruthenicum*. In contrast, the L group exhibited significantly longer roots and a higher germination rate than the CK group, suggesting that lower concentrations of NaCl treatment may promote the growth of *L. ruthenicum*.

Root ion profiles provided a second line of evidence. Quantification of Na^+^, K^+^, and Ca^2+^ showed pronounced divergence among treatments (one-way ANOVA, Figure 1d), with one exception: total Ca^2+^ remained statistically unchanged between the low- and high-salt regimes (Figure 1e). These soluble-ion signatures not only mirror the functional state of the membrane system but also gauge the intensity of the imposed stress. However, the Ca^2+^ pattern parallels the contrasting phenotypes: the modest salt dose that boosted germination (L group) left Ca^2+^ levels unaltered, whereas the inhibitory high dose (H group) failed to shift Ca^2+^ further, suggesting that Ca^2+^ balance is tightly linked to the threshold between stimulatory and suppressive salt stress.

### 2.2. Changes in Metabolites of Seed Initial Germination Under Salt Stress

**Figure 2:**
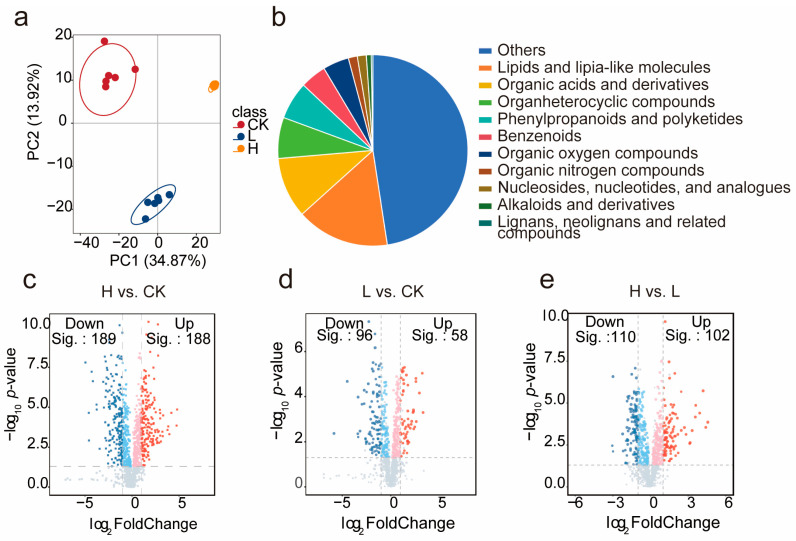
The analysis of metabolome: (**a**). The PCA analysis of 721 differential metabolites in 3 groups; (**b**). Composition of the identified compounds; (**c**). The volcano analysis of H vs. CK; (**d**). The volcano analysis of L vs. CK; (**e**). The volcano analysis of H vs. L.

Metabolite profiles from both control and salt-stress conditions were analyzed using UHPLC-MS. In the picture of Principal Component Analysis (PCA), it indicates that our samples exhibit substantial inter-group variability while maintaining strong intra-group consistency. Furthermore, hierarchical clustering confirmed the distinct grouping of samples by condition (Figure 2a).

In the experiment, 721 differentially accumulated metabolites (DAMs) were identified based on the criteria (|log_2_FC| > 0.5, *p*-value < 0.05, VIP > 1) (Table S1). And those DAMs were categorized into six major classes. These classes included lipids and lipid-like molecules (15.86%), organic acids and derivatives (10.43%), and phenylpropanoids and polyketides (6.26%) (Figure 2b). Among these, lipids were the largest group, encompassing subcategories such as fatty acids, triterpenoids, and terpene glycosides.

Volcano plot analysis revealed that metabolite expression varied significantly under severe salinity stress. In the H vs. CK comparison (Figure 2c), among the 377 DAMs identified, 188 were significantly upregulated and 189 were significantly downregulated. In the L vs. CK comparison (Figure 2d), 154 DAMs were detected. Fifty-eight DAMs were significantly upregulated, and 96 of them were significantly downregulated. As for the H vs. L comparison, 102 compounds were upregulated and 110 were downregulated (Figure 2e).

**Figure 3:**
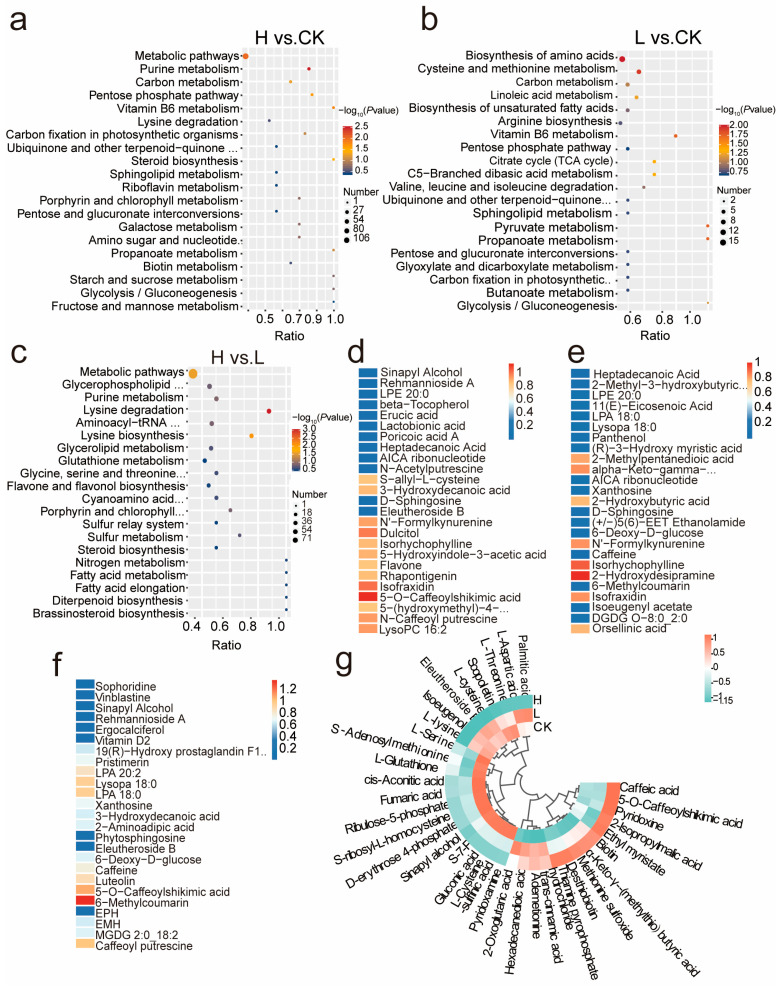
Metabolism analysis of 721 compounds. **a**–**c**. KEGG bubble plots of DAMs in *L. ruthenicum* between different groups: (**a**). Group H vs. CK; (**b**). Group L vs. CK; (**c**). Group H vs. L; (**d**–**f**). Heatmap which demonstrates the top 25 significantly differentially expressed metabolites in *L. ruthenicum* under different conditions; (**d**). Group H. vs. CK; (**e**). Group L vs. CK; (**f**). Group H. vs. L; (**g**). Clustering of 36 key compounds content among different treatments. The color of the blocks represents the abundance of the compound. Orange represents high levels of expression, and green represents low levels.

Kyoto Encyclopedia of Genes and Genomes (KEGG) enrichment analysis can also be used to reveal significant alterations in metabolic pathways of *L. ruthenicum* under salinity stress. In this analysis, general metabolic processes, amino acid metabolism, lipid metabolism, and secondary metabolite biosynthesis were highly enriched. In the H vs. CK (Figure 3a) comparison, specific enriched pathways included purine metabolism, carbon metabolism, pentose phosphate pathway, vitamin B6 metabolism, lysine degradation, and carbon fixation in photosynthetic organisms. In the L vs. CK comparison (Figure 3b), enriched pathways involved amino acid biosynthesis, cysteine and methionine metabolism, linoleic acid metabolism, and valine, leucine, and isoleucine degradation. Unique to the H vs. L comparison were pathways such as amino-acyl-tRNA biosynthesis, flavone and flavanol biosynthesis, fatty acid elongation, and sulfur metabolism (Figure 3c). Overall, salt stress activated pathways associated with amino acid metabolism, cofactors and vitamins, energy metabolism, and carbohydrate metabolism.

Furthermore, Figure 3d–f shows the top 25 DAMs across the three comparison groups (H vs. CK, L vs. CK, and H vs. L). In the H vs. CK group, metabolites such as benzenoids, lipids and lipid-like molecules, nucleosides, and organic nitrogen compounds were upregulated, while organic acids, oxygen compounds, organoheterocyclic compounds, and phenylpropanoids were downregulated. In the L vs. CK group, most lipids, nucleosides, organic nitrogen compounds, and specific metabolites like 6-deoxy-*D*-glucose, 6-methylcoumarin, and caffeine were increased, whereas a few compounds, such as *N*′-Formylkynurenine, decreased. In the H vs. L comparison, most metabolites were upregulated at higher NaCl concentrations, except for caffeoylputrescine, phenylpropanoids, and some lipids, which showed less pronounced changes.

To identify key metabolites, we calculated the correlation between different compounds using Pearson correlation analysis (|*r*| > 0.8 and *p* < 0.05). Based on that, several metabolites were selected for further examination and subsequently clustered into four classes (Figure 3g). For instance, compounds associated with cysteine and methionine metabolism (map00270), cutin, suberine, and wax biosynthesis (map00073), and pyruvate metabolism (map00620) pathway-related compounds, exemplified by L-cysteine, fumaric acid, were most abundant in group L, while those in the H group were low. These findings indicate that the activation of these pathways contributes substantially to *L. ruthenicum*’s growth promotion under mild salt stress. In contrast, proline was most abundant in group H, indicating that arginine and proline metabolism (map00330) and related pathways contribute to the plant defense under severe salt stress.

### 2.3. The Transcriptomic of L. ruthenicum Responding to Salt Stress

**Figure 4:**
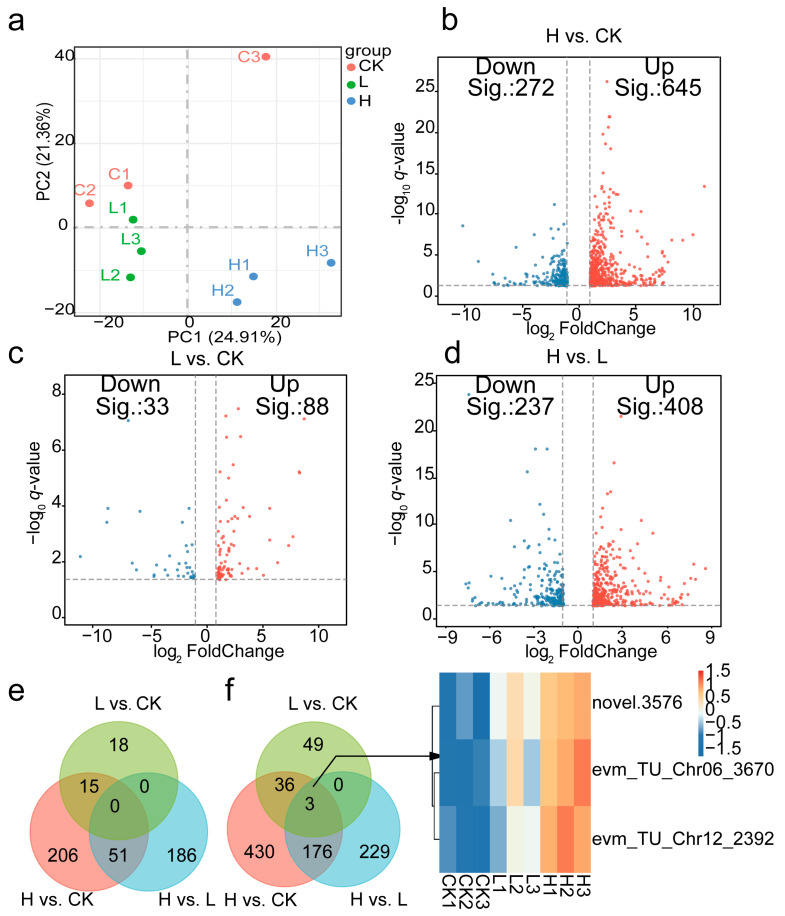
Transcriptome analysis of DEGs in *L. ruthenicum*: (**a**). PCA analysis of transcriptome analysis in *L. ruthenicum* among different conditions. (**b**–**d**). Volcano plot of transcriptome analysis in *L. ruthenicum* under different conditions. (**b**). H vs. CK; (**c**). L vs. CK; (**d**). H vs. L; (**e**). Venn diagrams of the downregulated genes among different comparisons in *L. ruthenicum*; (**f**). Venn diagrams of the upregulated genes and the content of 3 genes that existed in all comparisons in different treatments.

To explore the molecular mechanisms underlying *L. ruthenicum*’s response to salinity stress, transcriptomic approaches were employed to screen differentially expressed genes (DEGs) across various salinity conditions. High-quality RNA-seq data (64.7 Gb; Q30 ≥ 95.85%; GC content 42.11–42.93%) ensured reliable analysis, with over 73% of unique genes successfully annotated. As shown in Figure 4a, PCA distinguished treatment groups with high replicability. DEG analysis revealed 917 DEGs in the H vs. CK (645 upregulated DEGs, 272 downregulated DEGs), 121 in the L vs. CK (88 upregulated DEGs, 33 downregulated DEGs), and 645 DEGs in the H vs. L (408 upregulated, 237 downregulated DEGs). Figure 4e,f shows the downregulated and upregulated genes among the three comparisons via a Venn diagram. Notably, key genes such as lysine-tRNA ligase, casparian strip membrane protein 1 (CASP I), and early light-induced protein were identified, overlapping and unique DEGs were highlighted, revealing 55 shared DEGs between L vs. CK and H vs. CK, and 230 shared DEGs between H vs. L and H vs. CK. Additionally, only three genes were common across all three comparisons: *evm_TU_Chr06_3670*, *novel.3576,* and *evm_TU_Chr12_2392*, members of the MADS-box gene family (Figure 4f).

Gene ontology (GO) enrichment analysis provided deeper insights into the biological processes, cellular components, and molecular functions [17] affected under different salinity conditions. In the H vs. CK comparison, significant changes in biological processes were observed in photosynthesis, cellular glucan metabolic process, and fatty acid biosynthetic process. Alterations of cellular components were noted in the photosystem, thylakoid, and membrane protein complex. Changes of molecular function included transferase activity, glucosyltransferase activity, and oxidoreductase activity (Figure S1a). In the H vs. L comparison, significant changes in biological processes were observed in the cellular carbohydrate metabolic process, response to stress, and defense response. Alterations of cellular components were noted in the cell wall, mitochondrial membrane, and apoplast. Molecular Functions: Changes included heme binding, peroxidase activity, and antioxidant activity (Figure S1b). In the L vs. CK group, significant changes in biological processes were observed in the lipid biosynthetic process, aminoglycan metabolic process, and fatty acid biosynthetic process. Alterations of cellular components were noted in the photosystem, thylakoid part, and membrane protein complex. Changes of molecular functions included chitinase activity, channel activity, and hydrolase activity, acting on ester bonds (Figure S1c).

**Figure 5:**
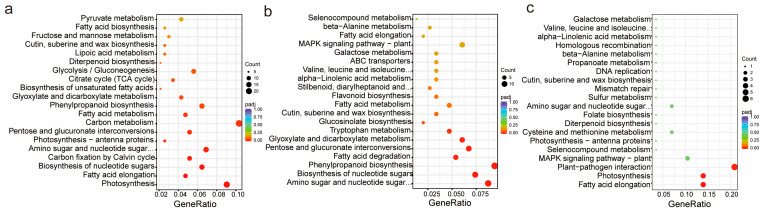
KEGG pathway analysis of DEGs in treated *L. ruthenicum* plants compared to the untreated control: (**a**). KEGG pathway analysis of H vs. CK group; (**b**). KEGG pathway analysis of H vs. L group. (**c**) KEGG pathway analysis of L vs. CK group. X axis represents the enrichment factor, and the Y axis represents the pathway name. The *Padj* value is demonstrated by the colors (high: blue, low: red); a smaller value indicates greater pathway significance. Point size indicates DEG number. Gene Ratio refers to the value of enrichment factor, which is the quotient of foreground value (the number of DEGs) and background value (total Gene amount). The value exhibits a positive correlation with significance.

KEGG pathway analysis further elucidated the metabolic pathways impacted by salinity stress. H vs. CK Comparison: Fifteen pathways were significantly enriched (*p* < 0.05), including glycolysis/gluconeogenesis, pyruvate metabolism, fatty acid biosynthesis, and fructose and mannose metabolism (Figure 5a), while photosynthesis and carbon fixation via the Calvin cycle were significantly upregulated (Figure S2a). L vs. CK Comparison: Significant enrichment was observed in pathways such as the MAPK signaling pathway, beta-alanine metabolism, and ABC transporters (Figure 5b), while fatty acid elongation, photosynthesis, and plant–pathogen interaction were upregulated significantly (Figure S3). H vs. L Comparison: Two pathways were significantly enriched (*p* < 0.05), including biosynthesis of nucleotide sugars and phenylpropanoid biosynthesis (Figure 5c). In comparison, the upregulation of them indicated that plants exhibit a more intense response to environmental stress under high-salinity conditions compared to low-salinity conditions. Conversely, the downregulation of ABC transporter pathways and MAPK signaling pathways suggested that the acquired salt tolerance mechanisms in *L. ruthenicum* under high salt stress, along with associated phenomena such as inhibited root growth, show no significant correlation with these two pathways (Figure S2b).

Besides the unique pathways identified in specific comparisons, such as glycolysis/gluconeogenesis in H vs. CK, tryptophan metabolism in H vs. L, and the MAPK signaling pathway in L vs. CK, six pathways were enriched across the comparisons. These shared pathways include biosynthesis of nucleotide sugars (lbb01250), amino sugar and nucleotide sugar metabolism (lbb00520), pentose and glucuronate interconversions (lbb00040), fatty acid metabolism (lbb01212), phenylpropanoid biosynthesis (lbb00940), and glyoxylate and dicarboxylate metabolism (lbb00630). Most of them were associated with primary metabolism.

### 2.4. KEGG Co-Enrichment Analysis of Differentially Expressed Genes and Metabolites in L. ruthenicum Under Salt Stress

In the H vs. CK group, pathways such as carbon metabolism, amino sugar and nucleotide sugar metabolism, and carbon fixation via the Calvin cycle, as well as glycolysis/gluconeogenesis and others, were enriched in both the metabolome and transcriptome. However, aside from amino sugar and nucleotide sugar metabolism, carbon metabolism and carbon fixation through the Calvin cycle, the other pathways were only significantly enriched in the transcriptome (*p* < 0.1). This suggested that these three pathways may play pivotal roles in the salt tolerance of *L. ruthenicum*.

A similar pattern was observed in the L vs. CK group. In this comparison, three pathways, cysteine and methionine metabolism, propanoate metabolism, and carbon metabolism, were significantly enriched in both omics analyses (*p* < 0.1). Meanwhile, other pathways such as sulfur metabolism, valine, leucine, and isoleucine degradation, galactose metabolism, ABC transporters, glyoxylate and dicarboxylate metabolism, and starch and mannose metabolism were only significantly enriched in the transcriptome. Functional enrichment analysis revealed that carbon metabolism was significantly enriched across both comparative groups, indicating substantial modulation by salt stress.

In the H vs. L group, lysine degradation was significantly enriched in both the metabolome and transcriptome (*p* < 0.1), while other pathways, including phenylpropanoid biosynthesis, fatty acid metabolism, and flavonoid biosynthesis, were only significantly enriched in the transcriptome. Interestingly, when considering the content of Ca^2+^, both carbon fixation via the Calvin cycle and carbon metabolism were identified in both H vs. CK and L vs. CK comparisons. These results suggested that the impact of salt stress on plants might be related to energy metabolism, especially carbon fixation via the Calvin cycle (ko00710).

**Figure 6:**
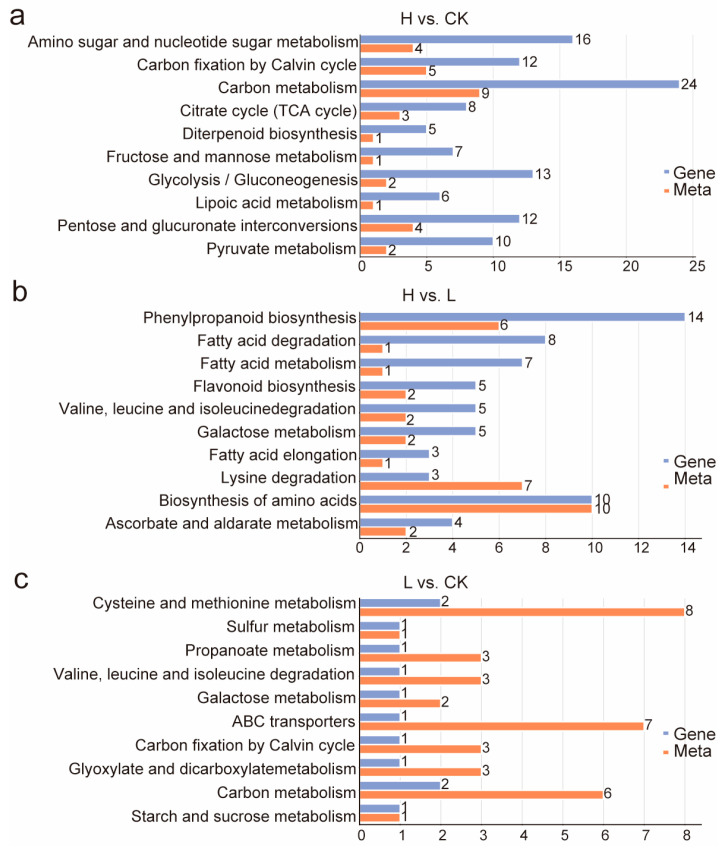
KEGG co-enrichment analysis for H. vs. CK. (**a**), H. vs. L. (**b**), and L. vs. CK (**c**). Horizontal axes indicate quantities of pathway-enriched differential metabolites and DEGs, the vertical axis represents the KEGG pathway names, and the orange and blue bars represent the metabolome and transcriptome, respectively.

KEGG co-enrichment analysis (Figure 6) identified five key metabolic pathways in this study: starch and sucrose metabolism (unique to L vs. CK), valine, leucine, and isoleucine degradation and galactose metabolism (enriched in both L vs. CK and H vs. L), along with photosynthetic carbon fixation and carbon metabolism (enriched in L vs. CK and H vs. CK). Among these five metabolic pathways, the photosynthetic carbon fixation pathway occupies a pivotal position, containing multiple metabolic nodes that can direct carbon flux to other pathways. Moreover, the key enzymes like RuBisCO (Ribulose-1,5-bisphosphate carboxylase/oxygenase) and RPIA (Ribose-5-phosphate Isomerase A) both play a vital role in response to energy metabolism and abiotic stress, especially salt stress. As shown in Figure S4, the expression levels of *novel.*5606, encoding a subunit of RuBisCO, and *rpiA*, were increased with rising salt concentrations, which were consistent with those obtained from RNA-Seq. This suggests that their expression changes are closely associated with the plant’s response to salt stress. Therefore, we propose that carbon fixation via the Calvin cycle is essential for seed germination and early growth of *L. ruthenicum* under salt stress.

### 2.5. Analysis of DEGs and DAMs Involved in the Carbon Fixation by Calvin Cycle (ko00710) Across Different Comparison Groups

Based on these findings, this section focuses on elucidating the variations in carbon fixation via the Calvin cycle between different comparison groups.

#### 2.5.1. Analysis of DEGs and DAMs in Carbon Fixation by Calvin Cycle in L vs. CK

**Figure 7:**
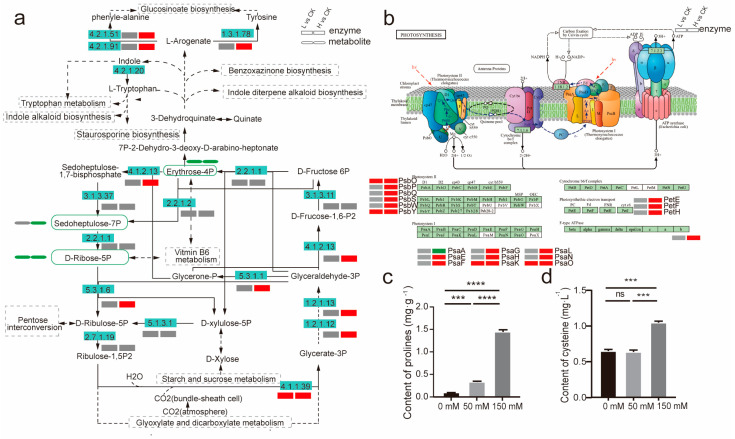
Assignment of a few key metabolites and genes to carbon fixation via the Calvin cycle: (**a**). Biological response pathway of *L. ruthenicum*’s seeds in response to salt stress. Analysis results of DEGs were mapped into a comprehensive metabolic regulation network diagram based on the KEGG pathway. (**b**). Changes existed in the enzymes’ expressed levels in the photosynthesis pathway. Red means a significant increase, green indicates a significant decrease, and gray means no significant change. (**c**). Proline content in *L. ruthenicum* plant. (**d**) Cysteine content in *L. ruthenicum* plant (*** *p* < 0.001, **** *p* < 0.0001, ns *p* > 0.05, *n* = 3). Red represents upregulated expression, and green represents downregulation; grey represents no significant change (*p* < 0.05). Solid lines denote a direct reaction, whereas dashed lines indicate that the conversion between two metabolites involves more than one step.

Based on integrative metabolic and transcriptome analysis, we proposed that sedoheptulose-7P may act as the key metabolite in the Calvin cycle because it was associated with erythrose-4P and *D*-ribose-5P. Additionally, erythrose-4P, as both its concentration and the level of downstream genes are altered under salt treatment. Related transcript abundance was upregulated while levels of sedoheptulose-7P, erythrose-4P, and D-ribose-5P were downregulated. To investigate the potential mechanisms underlying the accumulation of these compounds and the impact of metabolic pathways in *L. ruthenicum* seedlings under salt treatment, we reconstructed the relevant metabolic pathways (Figure 7a). In *L. ruthenicum* seedlings, the level of erythrose-4P and ribulose-5P was downregulated, while the transcript abundance of parts of RuBisCO’s encoding genes (*novel.5606*, *evm_TU_Chr12_2397*, *evm_TU_Chr12_2374*, and *evm_TU_Chr11_2375*) was upregulated under 50 mM NaCl treatment. However, the levels of most metabolites involved in starch and sucrose metabolism—which were direct indicators of abiotic stress, especially salt stress—remained unchanged. This indicated that low salt treatment may enhance the growth of *L. ruthenicum* seedlings by promoting photosynthesis (Figure 7b) and carbon fixation via the Calvin cycle, even though the plants already suffered oxidant damage, as evidenced by a significant increase in proline content (Figure 7c).

#### 2.5.2. Analysis of DEGs and DAMs in Carbon Fixation by Calvin Cycle in H vs. CK

In contrast to the L vs. CK, the levels of sedoheptulose-7P, erythrose-4P, and *D*-ribose-5P, involved in carbon fixation via the Calvin cycle, were downregulated under 150 mM NaCl treatment. Meanwhile, transcript abundance of *novel.5606*, encoding a subunit of RuBisCO, and the level of *rpiA* were upregulated with the decline in the level of expression of *psaA* (Figure S4). This change not only influenced PS I (Photosynthesis system I) functionality but also weakened the Calvin cycle by blocking the energy flux into it. Additionally, carbohydrate metabolism, such as starch and sucrose metabolism, was upregulated, accompanied by an increase in proline accumulation, which positively correlates with plant stress [7,30,31] (Figure 7c). The results suggested that high salt treatment may result in the inhibition of hypocotyl elongation during germination due to oxidant damage, even though major photosynthesis and carbon fixation via the Calvin cycle are particularly promoted.

### 2.6. Transcription Factor (TF) Analysis of the Study

Pearson correlation coefficient analysis revealed 241 DEGs, which are associated with all the enriched pathways and classified into 35 gene families. The top 10 gene families and the number of members in each are listed in Table 1, including *ERF*, *MYB*-related, *bHLH*, *C3H*, *B3*, *NAC*, *G2*-like, *MADS,* and *bZIP* families. Notably, across all comparisons, there are three genes (*evm_TU_Chr12_2397*, *evm_TU_Chr12_2374*, and *evm_TU_Chr11_2375*) involved in carbon fixation via the Calvin cycle (ko00710), and all of them are members of the M-type *MADS* family. Previous studies have revealed that these genes can enhance plant tolerance to abiotic stress.

Based on these findings, we proposed that *MADS* might be activated by salt stress, thereby promoting energy flow and carbon fixation through the utilization of related metabolites. Under low salt treatment, this mechanism appears beneficial, as it compensates for damage caused by mild environmental stress, particularly salt stress, and ultimately promotes plant growth. However, we inferred that when the level of stress increases, the benefits of enhanced photosynthesis are insufficient to counterbalance the negative effects of oxidative damage, or when photosynthesis is compromised by high salt stress, overall plant growth is hindered.

### 2.7. qRT–PCR

**Figure 8:**
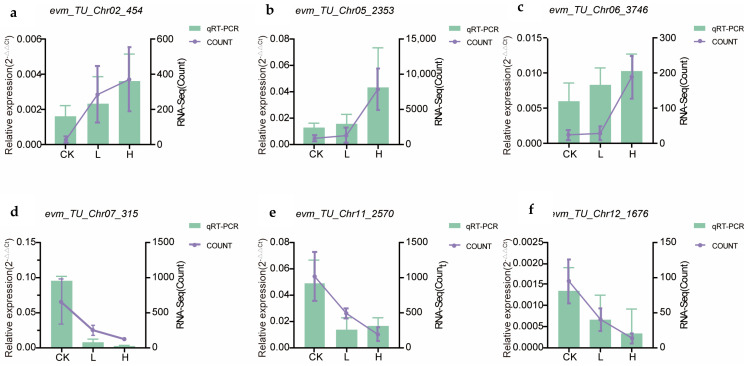
Six candidate genes were quantitatively assessed via qRT–PCR. (**a**). The quantitative results for gene *evm_TU_Chr02_454* align with the transcriptome data. (**b**). The quantitative results for gene *evm_TU_Chr05_2353* align with the transcriptome data. (**c**). The quantitative results for gene *evm_TU_Chr06_3746* align with the transcriptome data. (**d**). The quantitative results for gene *evm_TU_Chr07_315* align with the transcriptome data. (**e**). The quantitative results for gene *evm_TU_Chr11_2570* align with the transcriptome data. (**f**). The quantitative results for gene *evm_TU_Chr12_1676* align with the transcriptome data. The normalized expression data (Count values) are depicted as violet trendlines, aligned with the secondary (**right**) ordinate. In parallel, amplification efficiency was graphically represented through column diagrams referenced to the primary (**left**) Y-axis.

Considering their varied expression levels among different treatments, six DEGs were chosen for the qRT–PCR experiment to verify the accuracy of the RNA sequencing results. Genes that included *evm_TU_Chr02_454*, *evm_TU_Chr05_2353*, and *evm_TU_Chr06_3746* were highly expressed in L and H groups compared to the CK group, while *evm_TU_Chr07_315*, *evm_TU_Chr11_2570,* and *evm_TU_Chr12_1676* were expressed at low levels. qRT–PCR validation experiments confirmed the reliability of the transcriptomic data (Figure 8).

## 3. Discussion

*L. ruthenicum* is considered more economically important than wolfberry in sericulture. Salt stress, a significant abiotic factor impacting crop production, makes understanding the mechanisms behind seed germination responses crucial for enhancing crop tolerance [32]. For example, studies on alfalfa (*Medicago sativa* L.) have shown that root and hypocotyl growth significantly decrease as salt concentration increases [33,34]. To determine how salt stress influences the sprouting of *L. ruthenicum*, we conducted a series of seed germination experiments and measured various physiological indicators. In the present study, both the root length and germination rate of *L. ruthenicum* were significantly reduced under severe salt stress, as demonstrated by disrupted ion balance, altered root coloration, and changes in the potassium-to-sodium ratio. Specifically, *L. ruthenicum* seed sprouting was significantly inhibited under 150 mM NaCl treatment, while treatment with 50 mM NaCl promoted germination. That suggests we could improve the seeding of *L. ruthenicum* by adjusting the NaCl concentration in the environment. Seed germination is a critical growth stage that is highly sensitive to salt stress and involves complex interactions among multiple genes and metabolites [35].

As a functional food, most research on *L. ruthenicum* has focused on secondary metabolites such as flavonoids (which exhibit strong antioxidant activity) [7,35] and anthocyanidins (noted for their antioxidative properties and melanin inhibition) [36]. However, in addition to these compounds, *L. ruthenicum* plants also contain other metabolites such as erythrose-4P, sedoheptulose-7P, and D-Ribose-5P. These metabolites are part of the Calvin cycle, where the enzyme RPIA catalyzes the reversible conversion between ribose-5-phosphate and ribulose-5-phosphate. Previous studies have confirmed that PRIA plays a key role in energetic metabolism. Lu et al. also observed the upregulation of its coding genes during their investigation into the growth-promoting mechanism of salt treatment in *Nitraria sibirica* and proposed that the changes in gene expression levels might be regulated by Ethylene-responsive transcription factors (ERFs) [37]. This suggests that these metabolites may protect plants from salt stress by enhancing carbon fixation via the Calvin cycle.

Meanwhile, other researchers have investigated how *L. barbarum* responds to salt stress using phosphoproteomic analysis. Their findings suggest that salt tolerance may be attributed to accelerated conversion of intermediate metabolites and increased energy supply, driven by altered activities of key enzymes in the glycolytic pathway [38]. Furthermore, integrated transcriptomic and metabolomic approaches provide valuable insights into changes in metabolite levels and potential modifications in gene expression networks [39]. Using this approach, we conclude the role of *MADS* (including *evm_TU_Chr03_108*, *evm_TU_Chr12_2397*, *evm_TU_Chr12_2374*, and *evm_TU_Chr11_2375*) in regulating salt stress tolerance in *L. ruthenicum* seedlings during a seven-day seed germination period. RuBisCO, the rate-limiting enzyme of carbon fixation in the Calvin cycle, participates in the initial step of CO_2_ fixation [40] and is involved in *evm_TU_Chr12_2397*, *evm_TU_Chr12_2374*, and *evm_TU_Chr11_2375*. Under 50 mM NaCl treatment, the expression level of its encoding gene was significantly regulated, while others remained unchanged. At this lower salt concentration, the damage from stress was not severe enough to outweigh the benefits of enhanced photosynthesis, which may explain why low-salt treatment promotes seed germination. In contrast, under 150 mM NaCl, Calvin cycle metabolites were less abundant compared to those of the oxidative pentose phosphate pathway with the disruption of PS I (Photosynthesis system I) functionality through the downregulation of *psaA*. Maybe that is why the finding is consistent with previous research on sugar beet [41].

To validate the transcriptomic data, we performed quantitative real-time PCR (qPCR) verification on key target genes (Figure S4). As shown in Figure S4, the expression levels of *novel.5606* and *rpiA* were consistent with those obtained from RNA-Seq, showing that the expression change pattern of both genes increased with rising salt concentrations. Moreover, the downregulation of *psaA* in RNA-Seq and qRT–PCR both indicated that it may explain why high-concentration salt treatment inhibited the early growth of *Lycium ruthenicum.* All results confirmed that the data of the transcriptome are believable. Based on that, the alteration impaired Photosystem I (PSI) function, thereby hindering energy input into the Calvin cycle. Consequently, the reduced accumulation of downstream metabolites affected multiple biosynthetic pathways. These results suggest that photosynthesis, particularly the transcript abundance of *psaA* to regulate energy flux into the Calvin cycle, plays a key role in the biphasic response of *L. ruthenicum* seedlings to increasing salt concentrations. These findings offer a new perspective on how *L. ruthenicum* modulates its physiological and molecular responses under varying salt stress levels.

Although our results support a relatively clear mechanistic framework, the inherent specificity and limitations of omics technologies mean that further biochemical and functional validation (e.g., enzyme activity, Fv/Fm measurements) is required. This will be the focus of our next phase of research.

## 4. Materials and Methods

### 4.1. Plant Materials and Growth Conditions

Black wolfberry (*L. ruthenicum*) seeds were obtained from the *Lycium* germplasm nursery in Ningxia province, China. All seeds were surface sterilized with 5% sodium hypochlorite, rinsed three times, and then sown in germination boxes lined with moist filter paper in Petri dishes. After stratification treatment, the dishes were transferred to a growth chamber set to a 10-h light/14-h dark photoperiod at 25 °C; this day was designated as day 0. After 7 days, plants were moved to darkness for 48 h, and then collected for transcriptomic analysis and six replicates for metabolomic analysis, with three biological replicates. All samples were immediately frozen in liquid nitrogen and stored at −80 °C until further use.

### 4.2. Measurement of Physiological and Phenotype Indexes

#### 4.2.1. Length of Root

On day 7, all germinated seeds were photographed, and their lengths were measured using ImageJ (1.54j). Meantime, the germination rate is recorded [42].

#### 4.2.2. Ion and Proline Content Determination

Ion contents in roots and leaves were determined following the protocol of Weimberg [43] with modifications. Next, 0.05 g of dried plant sample was mixed with and heated in a water bath at 95 °C for 15 min. The extract was then filtered through a 0.45 µm filter (Gelman Laboratory, PALL Corporation, San Diego, CA, USA)). The concentrations of Na^+^, K^+^, and bioavailable Ca^2+^ were quantified using a Thermo Scientific (Waltham, MA, USA) iCAP 7200 ICP-OES. The determination of proline content was performed using the ninhydrin colorimetric method with a microplate reader FIexA-200(Hangzhou Allsheng Instruments, Co., Ltd, Hangzhou, China).

### 4.3. RNA Extraction, Library Preparation, and Sequencing

RNA integrity was assessed using the Bioanalyzer 2100 system (Agilent Technologies, Santa Clara, CA, USA). Messenger RNA was purified from total RNA using poly-T oligo-attached magnetic beads. After library quality control, libraries were pooled according to their effective concentrations and targeted data amount, then subjected to Illumina sequencing. In the process, *novel.5606* was defective. Because it contains the domain that codes for the RuBisCO unit without sequencing and was the 5606th gene identified in the screening pipeline, we named it *novel.5606*.

### 4.4. Quantitative Real-Time PCR (qRT–PCR)

To verify the accuracy and reliability of the transcriptome sequencing results, nine DEGs were selected for qRT–PCR analysis. RNA was extracted using RNAPrep Pure Plant Plus Kit (Polysaccharides and Polyphenolics-Rich) (Tiangen Biotech, Beijing, China), and cDNA was synthesized using HiScript™ III RT SuperMix for qPCR (+gDNA wiper) (Vazyme, Nanjing, China) according to the manufacturer’s instructions. Actin and EFIa were used as a reference gene [44], and the ChamQ Universal SYBR Green qPCR Master Mix (Vazyme, Shanghai, China) was used for qRT–PCR detection. Primers were listed in Table S2. Each sample was tested three times. Relative expression levels were calculated according to the 2^−ΔΔCT^ method [44].

### 4.5. Bioinformatics Analysis

Raw sequencing reads in FASTQ format were first processed using fastp for quality control; all downstream analyses were based on high-quality clean data. The reference genome and gene model annotation files were downloaded directly from the genome website. Paired-end clean reads were aligned to the reference genome using Hisat2 v2.0.5. Mapped reads of each sample were assembled using StringTie (v1.3.3b) in a reference-based approach. Gene expression levels were quantified with featureCounts v1.5.0-p3, and FPKM values were calculated based on gene length and read counts. Differential expression analysis was performed using the DESeq2 R package (1.20.0) with biological replicates. *p*-values were adjusted using the Benjamini and Hochberg method to control the false discovery rate, with thresholds set at a corrected *p*-value ≤ 0.05 and |log_2_ (fold change) | ≥ 1.

GO and KEGG enrichment analyses of differentially expressed genes were conducted using the clusterProfiler R package, which corrects for gene length bias. GO terms with a corrected *p*-value < 0.05 were considered significantly enriched, and KEGG pathway enrichment was similarly assessed (http://www.genome.jp/kegg/ (accessed on 10 November 2024)).

### 4.6. Metabolomics Sample Preparation and Data Processing

For metabolomic analysis, 100 mg of seeds was individually ground in liquid nitrogen. The homogenate was resuspended in prechilled 80% methanol and vortexed thoroughly. After incubation on ice for 5 min, samples were centrifuged at 15,000 g for 20 min at 4 °C. A portion of the supernatant was diluted with LC-MS grade water to a final concentration of 53% methanol, then transferred to a fresh Eppendorf tube and centrifuged again at 15,000 g for 20 min at 4 °C. The final supernatant was injected into the LC-MS/MS system for analysis [45].

The raw data files generated using UHPLC-MS/MS were processed using Compound Discoverer 3.3 (CD3.3, ThermoFisher (Waltham, MA, USA)) for peak alignment, peak picking, and quantitation. Metabolites were identified by matching the data with the mzCloud (https://www.mzcloud.org/ (accessed on 5 September 2024)), mzVault, and MassList databases. Statistical analyses were performed using R (R version R-3.4.3), Python (Python 2.7.6 version), and CentOS (CentOS release 6.6).

Metabolite annotation was conducted using the KEGG (https://www.genome.jp/kegg/pathway.html (accessed on 6 September 2024)), HMDB (https://hmdb.ca/metabolites), and LIPIDMaps (http://www.lipidmaps.org/) databases. PCA and PLS-DA were performed using metaX [46]. Univariate analysis (Student’s *t*-test) was applied to calculate statistical significance (*p*-value), and metabolites with VIP > 1 and *p*-value < 0.05 and |log2 (fold change)| ≥ 2 were considered significantly different. The functions of these metabolites and their associated metabolic pathways were analyzed, with pathways exhibiting *p*-values < 0.05 considered significantly enriched. Volcano plots were generated using ggplot2 in R, based on log_2_ (fold change) and –log_10_ (*p*-value). Co-enrichment and circular heatmap produced using the online platforms Bioinformatics.com.cn (last accessed on 10 November 2024) and Chiplot (https://www.chiplot.online), respectively. Bioinformatic analysis was performed using the OECloud tools (https://cloud.oebiotech.com).

## 5. Conclusions

In this study, we investigated the regulatory mechanism of *L. ruthenicum* seed germination under salt stress using comprehensive transcriptomic and metabolomic analysis. Our results revealed that carbon fixation via the Calvin cycle was a key pathway in this process. Under low salt conditions, *L. ruthenicum* seed germination was promoted, which coincided with the upregulation of *evm_TU_Chr12_2397*, *evm_TU_Chr12_2374,* and *evm_TU_Chr11_2375*, three members of the M-type *MAD*S-box gene family, which code the rate-limiting enzyme of the Calvin cycle, RuBisCO, without causing significant oxidative damage. This upregulation enhances carbon fixation and reduces carbon loss during related physiological processes, leading to improved performance [47]. In contrast, under high salt conditions, more severe oxidative damage was observed, which adversely downregulated *psaA* and caused substantial harm to the biological membrane system, despite the upregulation of RuBisCO’s encoding gene and *ripA*. This resulted in blocked energy flow and affected early plant growth [48]. These observations were further supported by changes in additional physiological parameters. Collectively, these data suggest that *MADS* transcription factor families are key regulators of carbon fixation during *L. ruthenicum* seed germination under salt stress. This study provides new insights into the mechanism by which key pathways, such as carbon fixation via the Calvin cycle, regulate *L. ruthenicum* seed germination under salt stress.

## Figures and Tables

**Table 1:** Transcription factors involved in carbon fixation via the Calvin cycle.

TFs Classification	Number	
	150 mM vs. Control	50 mM vs. Control
*FAR1*	25	8
*ERF*	45	5
*bHLH*	39	4
*bZIP*	18	4
*C3H*	28	4
*G2-like*	19	4
*NAC*	56	4
*B3*	21	3
*MYB _related*	38	3
*Trihelix*	24	1
*MADS*	24	2

## Data Availability

The datasets generated and analyzed during the current study are available in the Supplementary Information files. The BioProject number of the transcriptome data is PRJNA1231381.

## Supplementary Materials

The following supporting information can be downloaded at: https://www.mdpi.com/article/10.3390/ijms26157537/s1.

## Abbreviations

The following abbreviations are used in this manuscript:

DAMs	Differential metabolites
DEGs	Differentially expressed genes
ERFs	Ethylene-responsive transcription factors
GO	Gene ontology
KEGG	Kyoto encyclopedia of genes and genomes
*L. ruthenicum*	*Lycium ruthenicum*. Murr.
ROS	Reactive oxygen species
RPIA	Sedoheptulose-1,7-bisphosphatase
RuBisCO	Ribulose-1,5-bisphosphate carboxylase/oxygenase
TCA	Trichloroacetic acid
VIP	Variable importance in projection

## References

[1] Qin X., Yin Y., Zhao J., An W., Fan Y., Liang X., Cao Y. (2022). Metabolomic and transcriptomic analysis of *Lycium chinese* and, *L. ruthenicum* under salinity stress. BMC Plant Biol..

[2] Liu Z., Shu Q., Wang L., Yu M., Hu Y., Zhang H., Tao Y., Shao Y. (2012). Genetic diversity of the endangered and medically important *Lycium ruthenicum* Murr. revealed by sequence-related amplified polymorphism (SRAP) markers. Biochem. Syst. Ecol..

[3] Hongqiao L., Suyama A., Mitani-Ueno N., Hell R., Maruyama-Nakashita A. (2021). A Low Level of NaCl Stimulates Plant Growth by Improving Carbon and Sulfur Assimilation in *Arabidopsis thaliana*. Plants.

[4] Masepan N., Intarasit S., Panya A., Jungklang J. (2025). Low NaCl Concentrations Increase Cotyledon Growth in Chinese White Radish (*Raphanus sativus* L. *var. longipinnatus* Bailey) Seedlings via Aquaporin-Mediated Water Transport. Plants.

[5] Wang Z., Zhang W., Huang W., Biao A., Lin S., Wang Y., Yan S., Zeng S. (2023). Salt stress affects the fruit quality of *Lycium ruthenicum* Murr. Ind. Crops Prod..

[6] Ma Y.-J., Duan H.-R., Zhang F., Li Y., Yang H.-S., Tian F.-P., Zhou X.H., Wang C.M., Ma R. (2018). Transcriptomic analysis of *Lycium ruthenicum* Murr. during fruit ripening provides insight into structural and regulatory genes in the anthocya-nin biosynthetic pathway. PLoS ONE.

[7] Lin S., Zeng S.A.B., Yang X., Yang T., Zheng G., Mao G., Wang Y. (2021). Integrative Analysis of Transcriptome and Metabolome Reveals Salt Stress Orchestrating the Accumulation of Specialized Metabolites in *Lycium barbarum* L. Fruit. Int. J. Mol. Sci..

[8] Yu Y., Ni Y., Qiao T., Ji X., Xu J., Li B., Sun Q. (2022). Overexpression of VvASMT1 from Grapevine Enhanced Salt and Osmotic Stress Tolerance in *Nicotiana Benthamiana*. PLoS ONE.

[9] Huda K.M.K., Banu M.S.A., Garg B., Tula S., Tuteja R., Tuteja N. (2013). *OsACA6*, a P-type IIB Ca^2+^ATPase pro-motes salinity and drought stress tolerance in tobacco by ROS scavenging and enhancing the expression of stress-responsive genes. Plant J..

[10] Guo X., Niu J., Cao X. (2018). Heterologous Expression of *Salvia miltiorrhiza* MicroRNA408 Enhances Toler-ance to Salt Stress in Nicotiana benthamiana. Int. J. Mol. Sci..

[11] Chen B.-X., Fu H., Gao J.-D., Zhang Y.-X., Huang W.-J., Chen Z.-J., Yan S.-J., Liu J. (2022). Identification of Metabolomic Biomarkers of Seed Vigor and Aging in Hybrid Rice. Rice.

[12] Xue T., Liu S., Liu J., Yuan Y. (2023). Metabolomics Based on GC-MS Revealed Hub Metabolites of Pecan Seeds Germinating at Different Temperatures. BMC Plant Biol.

[13] Wang Z., Zhu C., Liu S., He C., Chen F., Xiao P. (2019). Comprehensive Metabolic Profile Analysis of the Root Bark of Different Species of Tree Peonies (*Paeonia Sect.* Moutan). Phytochemistry.

[14] Li Z., Cheng B., Yong B., Liu T., Peng Y., Zhang X., Ma X., Huang L., Liu W., Nie G. (2019). Metabolomics and Physiological Analyses Reveal β-Sitosterol as an Important Plant Growth Regulator Inducing Tolerance to Water Stress in White Clover. Planta.

[15] Reyes-Pérez J.J., Ruiz-Espinoza F.H., Hernández-Montiel L.G., De Lucía B., Cristiano G., Murillo-Amador B. (2019). Evaluation of Glycosyl-Hydrolases, Phosphatases, Esterases and Proteases as Potential Biomarker for NaCl-Stress Tolerance in *Solanum lycopersicum* L.. Varieties. Molecules.

[16] Liang X., Wang Y., Li Y., An W., He X., Chen Y., Shi Z., He J., Wan R. (2022). Widely-Targeted Metabolic Profiling in *Lycium barbarum* Fruits under Salt-Alkaline Stress Uncovers Mechanism of Salinity Tolerance. Molecules.

[17] Arif Y., Singh P., Siddiqui H., Bajguz A., Hayat S. (2020). Salinity Induced Physiological and Biochemical Changes in Plants: An Omic Approach towards Salt Stress Tolerance. Plant Physiol. Biochem..

[18] Abdel-Farid I.B., Marghany M.R., Rowezek M.M., Sheded M.G. (2020). Effect of Salinity Stress on Growth and Metabolomic Profiling of *Cucumis Sativus* and *Solanum Lycopersicum*. Plants.

[19] Wei S., Yang X., Huo G., Ge G., Liu H., Luo L., Hu J., Huang D., Long P. (2020). Distinct Metabolome Changes during Seed Germination of Lettuce (*Lactuca sativa* L.) in Response to Thermal Stress as Revealed by Untargeted Metabolomics Analysis. Int. J. Mol. Sci..

[20] Chen D., Yang Y., Niu G., Shan X., Zhang X., Jiang H., Liu L., Wen Z., Ge X., Zhao Q. (2022). Metabolic and RNA Sequencing Analysis of Cauliflower Curds with Different Types of Pigmentation. AoB Plants.

[21] Liu K., Xu S., Xuan W., Ling T., Cao Z., Huang B., Sun Y., Fang L., Liu Z., Zhao N. (2007). Carbon monoxide counteracts the inhibition of seed germination and alleviates oxidative damage caused by salt stress in Oryza sativa. Plant Sci..

[22] Chen L., Liu L., Lu B., Ma T., Jiang D., Li J., Zhang K., Sun H., Zhang Y., Bai Z. (2020). Exogenous Melatonin Promotes Seed Germination and Osmotic Regulation under Salt Stress in Cotton (*Gossypium hirsutum* L.). PLoS ONE.

[23] Wang Y., Jiang W., Li C., Wang Z., Lu C., Cheng J., Wei S., Yang J., Yang Q. (2024). Integrated transcriptomic and metabolomic analyses elucidate the mechanism of flavonoid biosynthesis in the regulation of mulberry seed germination under salt stress. BMC Plant Biol..

[24] Na Jom K., Frank T., Engel K.-H. (2011). A Metabolite Profiling Approach to Follow the Sprouting Process of Mung Beans (*Vigna radiata*). Metabolomics.

[25] Guo S., Klinkesorn U., Lorjaroenphon Y., Ge Y., Na Jom K. (2021). Effects of Germinating Temperature and Time on Metabolite Profiles of Sunflower (*Helianthus annuus* L.) Seed. Food Sci. Nutr..

[26] Pan J., Li Z., Dai S., Ding H., Wang Q., Li X., Ding G., Wang P., Guan Y., Liu W. (2020). Integrative analyses of transcriptomics and metabo-lomics upon seed germination of foxtail millet in response to salinity. Sci. Rep..

[27] Cheng B., Hassan M.J., Feng G., Zhao J., Liu W., Peng Y., Li Z. (2022). Metabolites Reprogramming and Na^+^/K^+^ Transportation Associated with Putrescine-Regulated White Clover Seed Germination and Seedling Tolerance to Salt Toxicity. Front. Plant Sci..

[28] Li C., Wang C., Cheng Z., Li Y., Li W. (2024). Carotenoid biosynthesis genes *LcLCYB, LcLCYE,* and *LcBCH* from wolfberry confer increased carotenoid content and improved salt tolerance in tobacco. Sci. Rep..

[29] Zhang Z., He K., Zhang T., Tang D., Li R., Jia S. (2019). Physiological responses of Goji berry (*Lycium barbarum* L.) to saline-alkaline soil from Qinghai region, China. Sci. Rep..

[30] Rejeb K.B., Abdelly C., Savouré A. (2014). How Reactive Oxygen Species and Proline Face Stress Together. Plant Physiol. Biochem..

[31] Wei T.-L., Wang Z.-X., He Y.-F., Xue S., Zhang S.-Q., Pei M.-S., Liu H.-N., Yu Y.-H., Guo D.-L. (2022). Proline Synthesis and Catabolism-Related Genes Synergistically Regulate Proline Accumulation in Response to Abiotic Stresses in Grapevines. Sci. Hortic..

[32] Hayat S., Hayat Q., Alyemeni M.N., Wani A.S., Pichtel J., Ahmad A. (2012). Role of proline under changing environments: A review. Plant Signal Behav..

[33] Chen Y., Wang J., Yao L., Li B., Ma X., Si E., Yang K., Li C., Shang X., Meng Y. (2022). Combined Proteomic and Metabolomic Analysis of the Molecular Mechanism Underlying the Response to Salt Stress during Seed Germination in Barley. Int. J. Mol. Sci..

[34] He F., Yang T., Zhang F., Jiang X., Li X., Long R., Wang X., Gao T., Wang C., Yang Q. (2023). Transcriptome and GWAS Analyses Reveal Candidate Gene for Root Traits of Alfalfa during Germination under Salt Stress. Int. J. Mol. Sci..

[35] Long R., Gao Y., Sun H., Zhang T., Li X., Li M., Sun Y., Kang J., Wang Z., Ding W. (2018). Quantitative proteomic analysis using iTRAQ to identify salt-responsive proteins during the germination stage of two Medicago species. Sci. Rep..

[36] Chen X., Zhao G., Li Y., Wei S., Dong Y., Jiao R. (2023). Integrative Analysis of the Transcriptome and Metabolome Reveals the Mechanism of Chinese Fir Seed Germination. Forests.

[37] Lu L., Wang Y., Chen Y., Zhu L., Wu X., Shi J., Chen J., Cheng T. (2025). Salt stimulates carbon fixation in the halophyte *Nitraria sibirica* to enhance growth. For. Res..

[38] Liang W., Zhang Z., Yao N., Wang B., Yu W., Zhu Q., Yang S., Zeng J., Wang L., Liang W. (2025). Glycolysis and signal transduction participate in *Lycium barbarum* in response to NaCl stress through protein phosphorylation. BMC Plant Biol..

[39] Zhao J., Li H., Yin Y., An W., Qin X., Wang Y., Li Y., Fan Y., Cao Y. (2020). Transcriptomic and metabolomic analyses of *Lycium ruthenicum* and *Lycium barbarum* fruits during ripening. Sci. Rep..

[40] Maheshwari C., Coe R.A., Karki S., Covshoff S., Tapia R., Tyagi A., Hibberd J.M., Furbank R.T., Quick W.P., Lin H.C. (2021). Targeted knockdown of ribulose-1, 5-bisphosphate carboxylase-oxygenase in rice mesophyll cells. J. Plant Physiol..

[41] Hossain M.S., Persicke M., ElSayed A.I., Kalinowski J., Dietz K.-J. (2017). Metabolite profiling at the cellular and subcellular level reveals metabolites associated with salinity tolerance in sugar beet. J. Exp. Bot..

[42] Xu N., Lu B., Wang Y., Yu X., Yao N., Lin Q., Xu X., Lu B. (2023). Effects of Salt Stress on Seed Germination and Respiratory Metabolism in Different *Flueggea Suffruticosa* Genotypes. PeerJ.

[43] Weimberg R. (1987). Solute adjustments in leaves of two species of wheat at two different stages of growth in response to salinity. Physiol. Plant.

[44] Harshitha R., Arunraj D.R. (2021). Real-time quantitative PCR: A tool for absolute and relative quantification. Biochem. Mol. Biol. Educ..

[45] Want E.J., Masson P., Michopoulos F., Wilson I.D., Theodoridis G., Plumb R.S., Shockcor J., Loftus N., Holmes E., Nicholson J.K. (2013). Global metabolic profiling of animal and human tissues via UPLC-MS. Nat. Protoc..

[46] Wen B., Mei Z., Zeng C., Liu S. (2017). metaX: A flexible and comprehensive software for processing metabolomics data. BMC Bioinform..

[47] Huan L., Xie X., Zheng Z., Sun F., Wu S., Li M., Gao S., Gu W., Wang G. (2014). Positive Correlation Between PSI Response and Oxidative Pentose Phosphate Pathway Activity During Salt Stress in an Intertidal Macroalga. Plant Cell Physiol..

[48] Lu X., Huan L., Gao S., He L., Wang G. (2016). NADPH from the oxidative pentose phosphate pathway drives the operation of cyclic electron flow around photosystem I in high-intertidal macroalgae under severe salt stress. Physiol. Plant.

