# Supplementary figures and images for: Biphasic Salt Effects on Lycium ruthenicum Germination and Growth Linked to Carbon Fixation and Photosynthesis Gene Expression
Source: Int J Mol Sci. 2025 Aug 4;26(15):7537. doi: 10.3390/ijms26157537 (PMC12347441; doi:10.3390/ijms26157537)

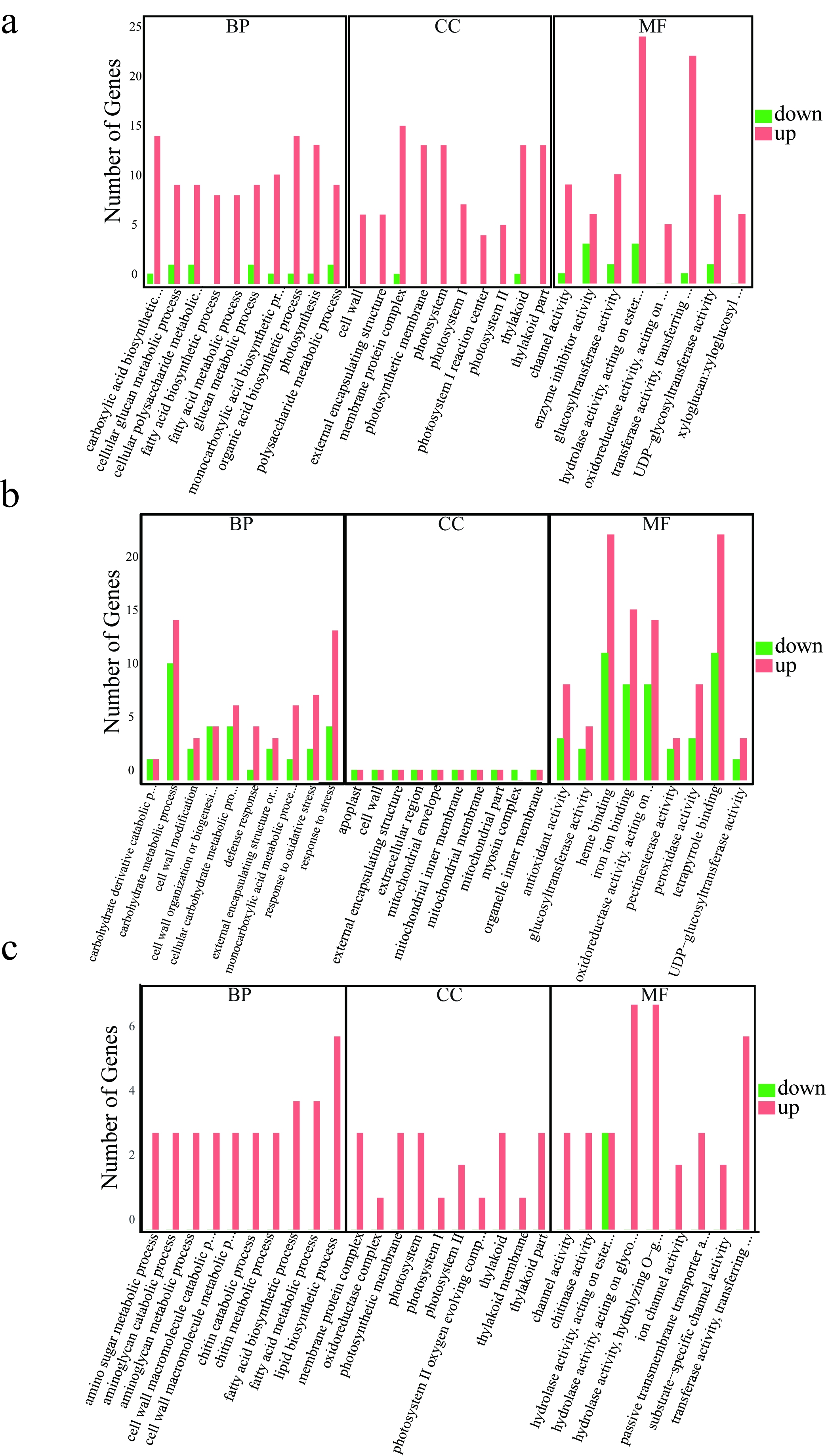

Supplement: Supplementary file 1 [file ijms-26-07537-s001.zip › Fig. S1.tif]

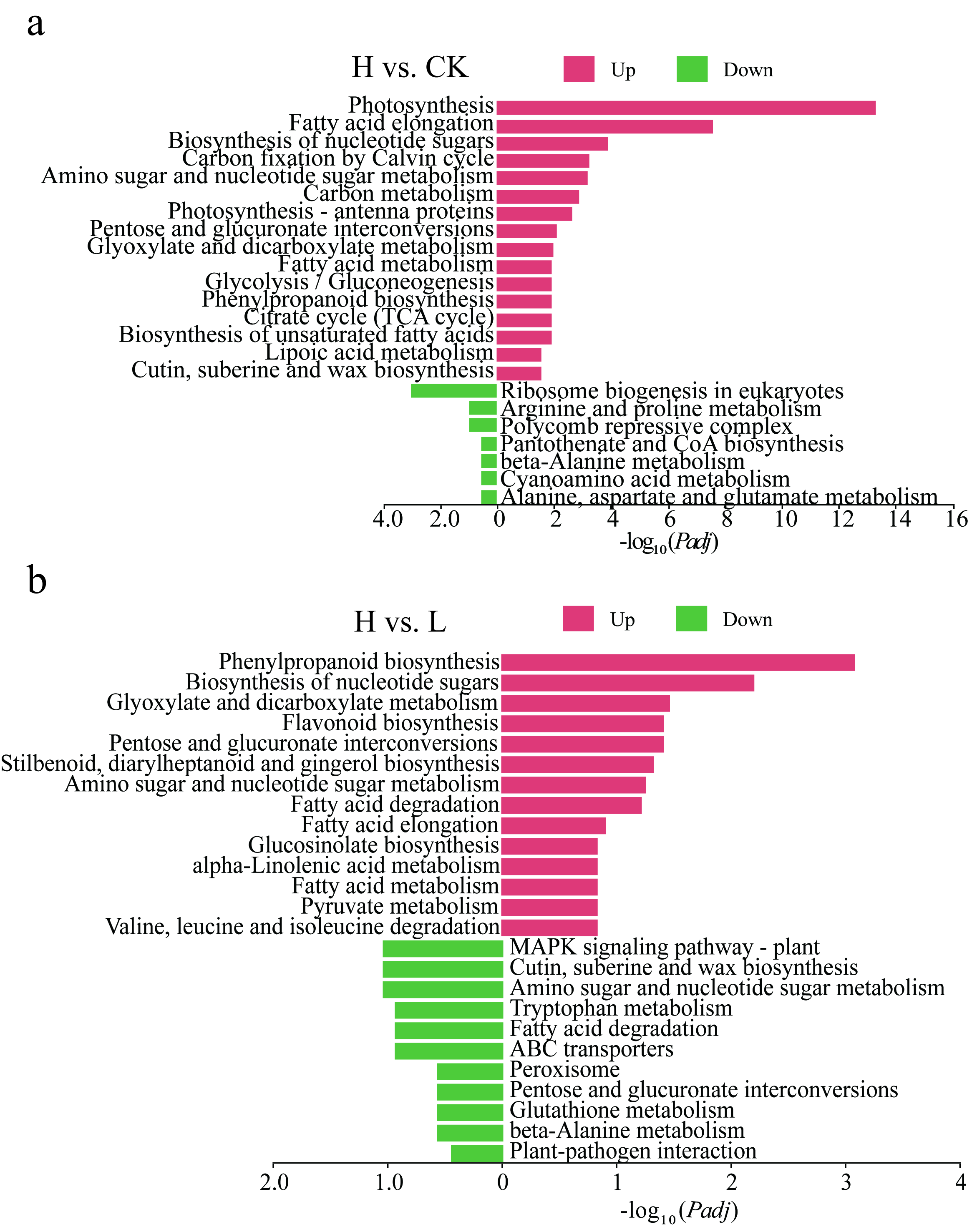

Supplement: Supplementary file 1 [file ijms-26-07537-s001.zip › Fig. S2.tif]

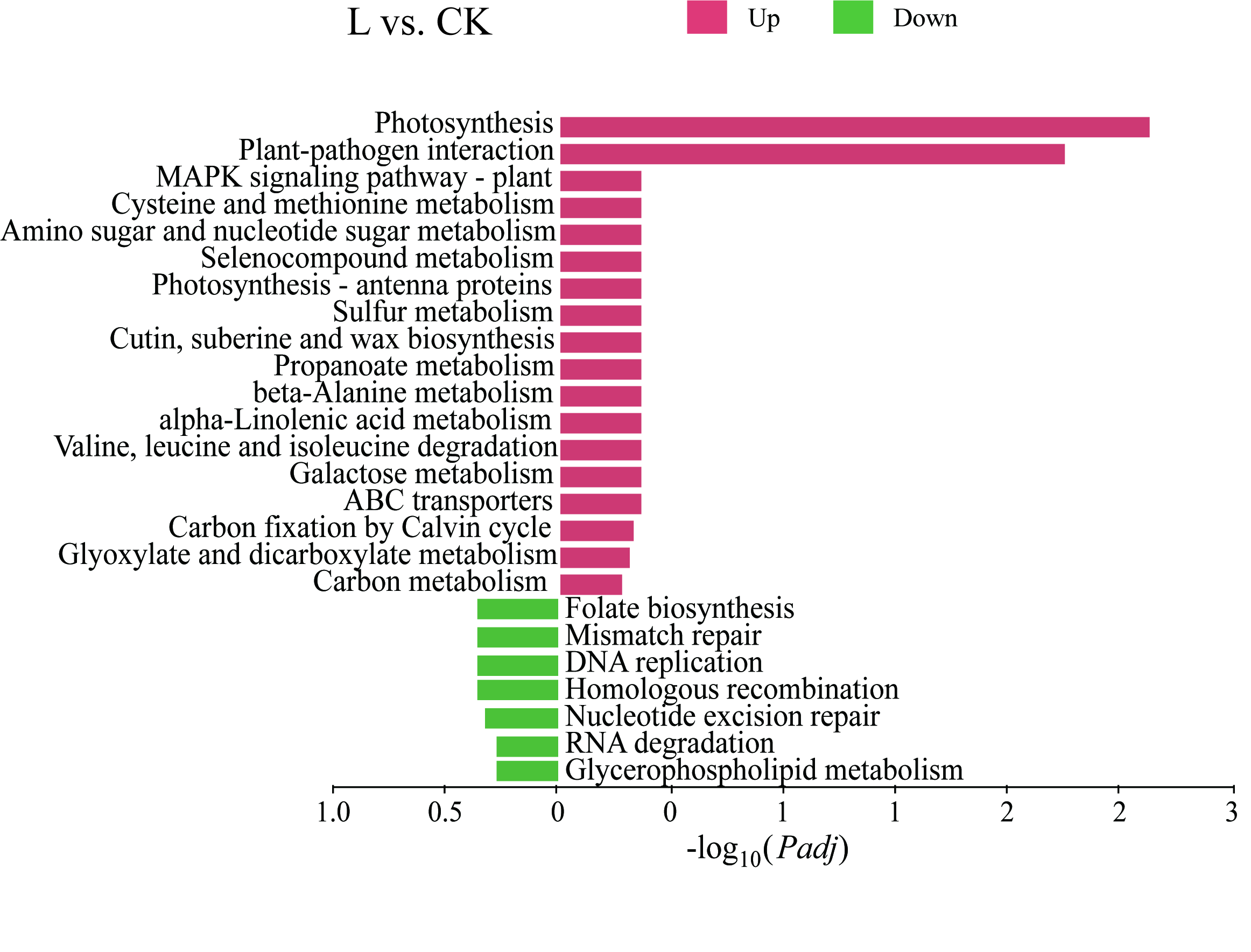

Supplement: Supplementary file 1 [file ijms-26-07537-s001.zip › Fig. S3.tif]

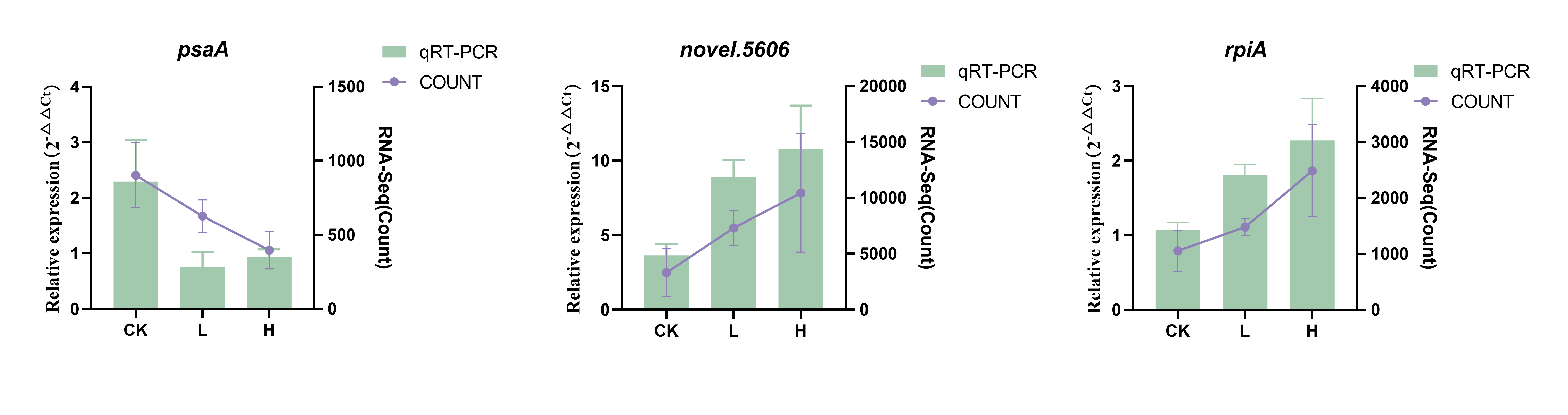

Supplement: Supplementary file 1 [file ijms-26-07537-s001.zip › Fig. S4.tif]
